# Oleamide, a Bioactive Compound, Unwittingly Introduced into the Human Body through Some Plastic Food/Beverages and Medicine Containers

**DOI:** 10.3390/foods9050549

**Published:** 2020-05-01

**Authors:** Katerina Naumoska, Urška Jug, Valentina Metličar, Irena Vovk

**Affiliations:** Department of Food Chemistry, National Institute of Chemistry, Hajdrihova 19, 1001 Ljubljana, Slovenia; urska.jug@ki.si (U.J.); valentina.metlicar@ki.si (V.M.)

**Keywords:** oleamide, leachables, extractables, food/beverages containers, medicine containers, polymer contact liquid

## Abstract

The purpose of the study was to investigate the migration of oleamide, a polymer lubricant, and a bioactive compound, from various plastic, marketed containers for food/beverages and medicines into polymer contact liquid. Methanol, food/medicine simulants or real samples were used to extract polymer leachables and extractables. Migrated oleamide into polymer contact liquids was determined by ultra-high performance liquid chromatography coupled to mass spectrometry (UHPLC-MS). The concentration of oleamide in the extracts of medicinal and insulin syringes was 7351 ng mL^−1^ and 21,984 ng mL^−1^, respectively. The leachates of intravenous (*i.v.*) infusion bottle, medicinal and insulin syringes contained 17 ng mL^−1^, 12 ng mL^−1^ and 152 ng mL^−1^, respectively. Oleamide in the extracts of dummies ranged from 30 to 39 ng mL^−1^, while in the leachates of baby bottles, from 12 to 23 ng mL^−1^. Leachates of soft drink bottles contained from 6 to 15 ng mL^−1^ oleamide, milk bottles from 3 to 9 ng mL^−1^, liquid yogurt bottles 17 ng mL^−1^ and water bottles from 11 to 18 ng mL^−1^. Bottled real matrices of oil and milk contained oleamide in the range from 217 to 293 ng mL^−1^. Moreover, the source of migrated oleamide (e.g., containers, caps, other parts) was identified. Oleamide is listed in the current EU regulations without a specific migration limit. Accordingly, these values are considered of no concern, unless future toxicological studies prove the opposite.

## 1. Introduction

Plastic materials are nowadays inevitable in human life due to their practical and convenient use. People are exposed daily to plastic containers used as food, beverage or medicine [1] packaging or delivery systems. Many additives are responsible for enhancing polymer properties and prolonging its shelf-life, thus increasing the functionality of the polymer products [2]. These additives can potentially contaminate water, soil and air and can migrate into end-user products from polymer contact materials, thus leading to human exposure [2]. Lubricants as additives are added to the plastic material in amounts from 0.1% to 3% *w*/*w* [2] in order to achieve better mechanical properties of the polymer, such as lubrication, prevention of film stickiness and antistatic properties [3,4]. Oleamide (*cis*-9-octadecenamide) is a well-known lubricant and slip additive from the group of fatty acid amides, besides erucamide ((*Z*)-docos-13-enamide) and stearamide (octadecanamide) [3,4].

Extractables and leachables are receiving much attention nowadays from the scientific public and regulatory bodies [5]. Public awareness was particularly raised by the case of the endocrine disruptors: bisphenol A, leaching from polycarbonate beverage containers, and phthalates, such as di(2-ethylhexyl) phthalate (DEHP) with potential carcinogenic [6] and estrogenic effects [7]. The concerns for the safety and toxicity of the end-user products (either pharmaceutical or food) have become major issues [8]. Oleamide was also detected in different plastic materials by several studies [3,9,10]. Migration of oleamide from the polymer is faster in comparison to erucamide, since it contains fewer carbon atoms [3], thus leading to higher human exposure.

Oleamide (an amide of oleic acid) is also an endogenous bioactive signaling molecule that acts in diverse cell types and consequently triggers different biological effects. Among the broad variety of functions, the most recognized one is its sleep-inducing effect [11,12]. Oleamide is synthesized in neurons and accumulates in the cerebrospinal fluid of sleep-deprived animals [11,12]. Induction of sedation and physiological sleep, when administered to animals, might be due to alteration of sleep affecting and cognitive processes related to cannabinergic CB-1 (cannabinoid receptor type 1) [13], serotonergic 5-HT (5-hydroxytryptamine receptors) or GABAA (gamma-aminobutyric acid type A) receptors [14,15]. Since nervous and immune systems are often cross-regulated, the potential immunoregulatory activity of oleamide was also studied and its anti-inflammatory effect has been proven [16,17,18,19,20]. Oleamide inhibits the proliferation of lymphocytes that play an important role in the specific immune response [16]. Activation of the NF-κB (nuclear factor kappa-light-chain-enhancer of activated B cells) transcription factor by LPS (lipopolysaccharide) in BV2 murine microglia was inhibited by oleamide. Consequently, the formation of pro-inflammatory mediators such as NO (nitric oxide) and prostaglandin E2 was inhibited, while expression of iNOS (inducible nitric oxide synthase) enzyme and COX-2 (cyclooxygenase-2) receptor, which are important proteins in the regulation of the immune system, was attenuated [17]. Oleamide, isolated from green algae *Codium fragile*, also suppressed LPS-induced expression of iNOS and COX-2, secretion of TNF-α (tumor necrosis factor-α), IL-1β (interleukin-1 beta) and IL-6 (interleukin 6) and prevented translocation of NF-κB into the nucleus. Carrageenan-induced rat paw edema was suppressed two hours after oleamide intraperitoneal injection, which was comparable to the effect of the nonsteroidal anti-inflammatory drug diclofenac [18]. By reduction of histamine and β-hexosaminidase secretion and by inhibition of interleukin-4 (IL-4) and TNF-α production, oleamide isolated from *Arctium lappa* extract showed anti-allergic potential in the early- and the late-phase response of the organism to allergen exposure [19]. Oleamide could be used for the treatment of inflammatory diseases connected with abnormal expression or activation of Toll-like receptor 2 (TLR2) and 4 (TLR4) and their adapter proteins, especially for peripheral inflammatory disorders, such as atopic dermatitis [20]. There are numerous other oleamide actions described such as cannabinoid-like behavior [13,21], inhibition of human monoamine oxidase B enzyme (hMAO B) [15,22], activation of TRPV1 (transient receptor potential vanilloid subtype 1) receptors [23], closure of gap-junctions [23], etc. Some of these mechanisms have an influence on the cardiovascular system [23]. 

Due to the fact that oleamide was found to be leaching from some plastic and other labware by our previous study [24], its presence in polymeric containers for food/beverages and medicines and its consequent leaching into the polymer contact liquid was assumed. Migration of oleamide from polymer starting materials used in food packing [9,25,26,27], food storage containers [28,29] or plastic baby bottles [10] into some food simulants was already reported. Oleamide leaching from baby bibs has also been assessed [30]. Personal and healthcare products such as, sanitary articles, sterile gauze, bandages, nappies and plasters were analyzed and oleamide was identified only in the sticking plaster [31]. Taking into account the limited quantitative data on oleamide leaching from food/medicinal containers into the consumer products found on the market, the scope of this paper was to study the migration of oleamide from these containers into polymer contact liquid (methanol, food/formulation simulants or real samples). For this purpose, the applicability of a quantitative UHPLC-MS method was examined and the possible oleamide intake into the human body was calculated. Moreover, the container components were tested separately to identify the source of migrated oleamide.

## 2. Materials and Methods

### 2.1. Chemicals and Materials

All solvents used in the study were at least of analytical grade. Methanol (liquid chromatography-mass spectrometry (LC-MS) grade) and ammonium bicarbonate were purchased from Honeywell Reagents (Seelze, Germany), ethanol was obtained from Carlo Erba (Val de Reuil Cedex, France), *n*-hexane was acquired from Merck (Darmstadt, Germany), ammonia solution (32%) was obtained from Sigma-Aldrich and ultrapure water (18 MΩ^−1^ cm) was supplied by a Milli-Q water purification system (Millipore, Bedford, MA, USA). Standard oleamide (≥99%) was purchased from Sigma-Aldrich (St. Louis, Missouri, United States). Polytetrafluoroethylene (PTFE) membrane filters (0.1 µm), used for mobile phase filtration, were acquired from Merck (Millipore, Carrigtwohill, Ireland). Hydrophilic polytetrafluoroethylene (H-PTFE) syringe filters were obtained from Macherey-Nagel (Duren, Germany). The use of plastic labware was avoided throughout the study and only pre-tested and oleamide-free labware was used to prevent false-positive results for oleamide. Besides pre-tested, oleamide-free H-PTFE membrane syringe filters, enclosed in a plastic housing, glass labware was exclusively used during the execution of the study. This included the use of a pre-tested, oleamide-free 10 mL glass syringe (Popper & Sons, New Hyde Park, New York, USA), a 100 μL glass syringe with a needle (Hamilton, Bondauz, Switzerland) and glass Pasteur pipettes (Brand, Wertheim, Germany). Binder incubators (Tuttlingen, Germany) were used to expose the containers to contact conditions.

### 2.2. Preparation of Extracts of Medicinal Plastic Materials

Medicinal plastic materials listed in Table 1 were extracted with methanol or formulation/food simulants, which were selected according to the document ‘Commission Regulation (EU) No 10/2011 of 14 January 2011 on plastic materials and articles intended to come into contact with food’ [32]. The intravenous infusion bottle was emptied, cleaned with warm and distilled water and filled with 20 mL of formulation simulant (10% ethanolic_(aq)_ solution), followed by exposure to contact conditions for 10 days in a dark environment at 60 °C. It was exposed to contact conditions to simulate its long-term contact with the formulation. The obtained extract after exposing to contact conditions was further dried under nitrogen and re-dissolved in methanol to obtain a 20-fold concentration. Other listed materials were not exposed to contact conditions as they are intended for single use only. Medicinal 5 mL syringes (also used in analytical laboratories for filtration) and 1 mL insulin syringes were rinsed with methanol or formulation simulant (H_2_O), while enteral syringes were rinsed by methanol or food simulant (50% ethanolic_(aq)_ solution) (Table 1). Some of the obtained extracts were analyzed by the LC-MS method (Section 2.4) without further pretreatment steps (label ‘non conc.’, Table 1), while the rest were 10-fold concentrated (label ‘conc.’, Table 1).

### 2.3. Preparation of Extracts of Baby Bottles and Plastic Food/Beverages Containers

Extracts of plastic baby bottles, baby bottle dummies and different plastic food/beverages containers listed in Table 1 were prepared for analysis by the LC-MS method (Section 2.4). Part of the samples was prepared by extraction with methanol (baby bottle dummies) or food simulants (baby bottles, bottles of carbonated soft drinks, vitamin water, milk, liquid yogurt and oil) (label ‘FS’, Table 1), which were selected according to the European Commission’s document [32]. The rest of them (bottled milk, oil and water) were analyzed as real samples (label ‘RS’, Table 1) after concentration in the case of water or following liquid-liquid extraction (LLE) in case of milk and oil, as described below. According to the European Commission’s document [32], the original vegetable oil is considered as a food simulant. Therefore, despite the same content, one bottled vegetable oil sample was considered as a real sample, while the other was taken as a food simulant (Table 1). Finally, all samples, except dummies methanolic extracts, bottled milk and bottled vegetable oil (the last two samples were analyzed as real samples) were exposed to contact conditions (Table 1).

Bottled water and bottled vegetable oil were exposed to 10 days of contact time at 60 °C contact temperature. Bottles of soft drinks and vitamin water were emptied, washed with warm and distilled water, filled with 3% acetic acid_(aq)_ as food simulant and exposed to contact conditions for 10 days at 60 °C. Bottles of milk and yogurt were also emptied, washed with warm and distilled water, filled with 50% ethanolic_(aq)_ solution as food simulant and exposed to contact conditions for 10 days at 20 °C. Sterilized baby bottles cleaned with boiling and distilled water were filled with 50% ethanolic_(aq)_ solution as food simulant and exposed to contact conditions for 2 h at 40 °C. Contact conditions were selected according to Annex V, Chapter 2 of the European Commission’s document [32]. Since the appropriate temperature of the infant formula is the body temperature and it is used within two hours of preparation, recommended contact conditions at 40 °C for 2 h were selected, thus simulating its storage in the baby bottles of a maximum of 2 h at body temperature [32]. Considering the fact that *i.v.* infusion (Section 2.2), soft drink, vitamin water, oil and water are in contact with the container for a long period (more than 6 months) and are usually stored at room temperature, the recommended contact conditions at 60 °C for 10 days were chosen, thus simulating their long-term storage in the containers above 6 months at room temperature or below [32]. Milk and yogurt usually spend less time (up to 30 days) in contact with the containers and are stored in the fridge. Therefore, the recommended contact conditions at 20 °C for 10 days were selected to simulate their contact in the fridge from 3 to 30 days [32]. After exposing to contact conditions, the solvents of all extracts, except those corresponding to the two oil samples and a milk sample, were evaporated under nitrogen and the solid residues were re-dissolved in methanol, thus obtaining 2-fold concentration for storage at –20 °C and 20-fold concentration (label ‘conc.’, Table 1) for LC-MS analysis.

To determine oleamide leached and/or present in the complex matrices, such as vegetable oil and milk, LLE for oil (both exposed and non-exposed to contact conditions) and milk (non-exposed to contact conditions) samples was performed (Table 1). LLE for oil was done by shaking 20 mL of the oil and 10 mL of methanol in a separatory funnel for 2 min. The upper methanolic layer was collected and filtered through a 0.2 μm H-PTFE filter attached to a glass syringe. LLE for milk was performed by shaking 20 mL of the milk and 10 mL of *n*-hexane in a separatory funnel for 2 min. After 5 min of equilibration of the phases, the upper *n*-hexane phase was transferred by a glass pipette to another separatory funnel, where an additional 10 mL of *n*-hexane was added. After shaking for 2 min and equilibrating the phases for 5 min, the upper clear layer was taken and filtered through a 0.2 μm H-PTFE filter. The solvent of the extract was evaporated under nitrogen and dry residues were reconstituted in methanol, thus obtaining 2-fold concentration. The extract was further kept in a freezer for 2 h. The obtained milk methanolic extract was again filtered through 0.2 μm H-PTFE before its LC-MS analysis. Additionally, oil (both exposed and non-exposed to contact conditions) and milk (non-exposed to contact conditions) samples were spiked with oleamide standard to obtain its final concentration of 25 μg mL^−1^ in the LLE extraction solvent. For calculation of the recovery (%) of oleamide obtained after LLE, the amount of oleamide in the non-spiked extracts was subtracted from the amount of oleamide in spiked extracts and the result was divided by the amount of oleamide standard with a concentration of 25 μg mL^−1^.

Baby bottle dummies were soaked in 20 mL of methanol in a glass beaker for 5 min and the extract was concentrated 10-fold (Table 1).

### 2.4. LC-MS Analysis

Samples, prepared according to the protocols described in Section 2.2 and Section 2.3 and transferred to HPLC (high-performance liquid chromatography) vials using a pre-tested, oleamide-free 100 μL glass syringe with a needle, were analyzed for oleamide by the UHPLC-MS method using the UHPLC-MS system (Dionex Ultimate 3000—LCQ Fleet, Thermo Scientific, San Jose, CA, USA). This method was initially developed for the identification of oleamide in labware extracts (the first part of our study [24]), and further applied for its identification and quantification in food/beverages and medicine containers extracts (the second part of the study, current manuscript). Acquity UPLC BEH C18 column (100 × 2.1 mm; 1.7 µm, Waters Corporation Milford, MA, USA) attached to a C18 guard column (4 × 2 mm, Phenomenex) was used for separation of oleamide and other polymer extractables and leachables. The mobile phase was composed of 10 mM ammonium bicarbonate buffer with pH 10.77 (A) and methanol (B) (20:80, *v*/*v*). The buffer was prepared by dissolving 0.79 g ammonium bicarbonate in 1 L Milli-Q water. Ammonia solution was used to adjust the pH of the buffer solution. Prepared mobile phase solvents were separately filtered through a 0.1 µm PTFE membrane filter. Isocratic elution was employed using a flow rate of 0.2 mL min^−1^. Tray and column temperatures were maintained at 10 and 30 °C, respectively [24]. The run time was 30 min or longer (for analysis of oil and milk extracts obtained after LLE, Section 2.3). In the latter case, cleaning step including gradient elution using Milli-Q water (A) and methanol (B): 80%–90% B (30–35 min), 90% B (35–50 min), 90%–80% B (50–55 min), followed by re-equilibration step using 10 mM ammonium bicarbonate buffer pH 10.77 (A) and methanol (B): 80% B (55.01–60 min), was applied. 

Heated electrospray ionization (HESI) in positive ion mode was used for compounds’ ionization. Oleamide methanolic standard solution (25 μg mL^−1^) was employed to tune the MS parameters. Heater and capillary temperatures were maintained at 150 and 350 °C respectively, flow rates of the sheath, auxiliary and sweep gases were set at 19 a.u. (arbitrary units), 10 a.u. and 0 a.u. respectively, spray and capillary voltages were optimized to 4.00 kV and 23.41 V respectively, and tube lens value was set at 91 [24]. The range of *m*/*z* 100–1000 was chosen for total ion chromatogram (TIC) mass spectra recording. Oleamide (*m*/*z* 282) was also monitored using selected ion monitoring (SIM) chromatograms. For multistage MS (MS^n^) experiments, collision energy of 30% was used for the fragmentation of oleamide protonated molecules (precursor ions).

For quantification of oleamide, standard solutions with 12 different concentrations of oleamide in μg mL^−1^ range (0.024, 0.049, 0.098, 0.195, 0.391, 0.781, 1.563, 3.125, 6.25, 12.5, 25 and 50 were prepared and SIM mode was used. Using these standard concentrations, two calibration curves were plotted. Thermo Xcalibur software (version 4.0.27.42) was employed for the acquisition and processing of the obtained data. 

Limit of detection (LOD), limit of quantification (LOQ), % relative standard deviation (RSD) of the areas of oleamide standard peak at 25 µg mL^−1^ (*n* = 6), R^2^, range, accuracy and precision were determined. The LOD and LOQ were calculated as concentrations of oleamide (*n* = 6), resulting in a signal-to-noise ratio of at least 3 and 10, respectively. Due to the relatively simple matrix of the extracts and leachates, dried (under nitrogen) and re-dissolved in methanol for LC-MS analysis, accuracy was calculated as % of the recovered amount from the spiked blanks (*n* = 3) at 0.391 µg mL^−1^ and at 6.25 µg mL^−1^ for the first and second curve, respectively. Precision was determined as % RSD (areas) measured using the vitamin water sample (representative for aqueous samples) and oil sample, non-exposed to contact conditions (representative for fatty samples), prepared in six replicates.

### 2.5. Statistical Analysis

Paired t-test was performed using Microsoft Excel 2010 (Microsoft Corporation, Redmond, Washington, USA) to test the statistical significance of the results (*n* = 3). *p* < 0.05 was considered significant.

### 2.6. Identification of the Source of Migrated Oleamide

The items that showed migration of oleamide above LOQ (Table 1) were further tested to identify its source. Each item component that had the potential to come into contact with drugs or food/beverages was considered separately. These parts include barrels and plungers for medicinal syringes, containers and dummies for baby bottles, containers and closures (caps) for *i.v.* infusion and food/beverages bottles. Each of these items had only two components potentially exposed to contact liquid, except oil bottle, which was a three-component system (container and closure system comprised of a cap and an intermediate part located on the neck of the bottle and directly below the cap).

All items were emptied and cleaned as explained in Section 2.2 and Section 2.3. Care was taken to clean all components separately. Barrels of the medicinal syringes were filled with methanol to the uppermost mark. To help keep the liquid inside, a pre-tested oleamide-free filter was mounted on the needle adapter, although the extract was not filtered through the mounted filter afterward. The parts of the syringe plungers that are intended to come into contact with the syringe barrel were dipped into 8 mL methanol in a narrow glass beaker. Containers, separated from the caps, were filled with 20 mL methanol, caps were filled with 4 mL methanol, while the intermediate component of the oil bottle was unmounted from the container and dipped into 20 mL methanol using a glass beaker. Contact with the solvent of 5 min was allowed for all components. The extracts were further dried under nitrogen and re-dissolved in methanol to obtain a 20-fold concentration of the extracts. The LC-MS method in SIM mode, described in Section 2.4, was used for the identification of oleamide in the prepared extracts.

## 3. Results and Discussion

### 3.1. LC-MS Method

Oleamide was separated from other polymer leachables and extractables using an isocratic UHPLC method. For analysis of complex matrices, such as oil and milk extracts, column cleaning and re-equilibration steps were introduced (Section 2.4). The influence of 0.1% formic acid, 0.1% ammonia solution_(aq)_ and 10 mM ammonium bicarbonate buffer (pH 10.77) as weak solvents combined with methanol on the chromatography of oleamide was tested. The addition of 0.1% formic acid into the mobile phase resulted in a broad and distorted chromatographic peak. By using 0.1% ammonia solution_(aq)_ instead, the peak for oleamide in TIC was of low intensity and the peaks of other plastic migrants were not observed. Finally, ammonium bicarbonate buffer (10 mM, pH 10.77) as a weak solvent showed best chromatographic performances (good peak shape, repeatable retention times, baseline separation) and provided the best sensitivity for oleamide and other migrants originating from the plastic syringe. 

The optimized MS method for oleamide standard employing HESI in positive ion mode was used for compound ionization and spectra acquiring. The MS spectrum of oleamide showed a protonated molecule at *m*/*z* 282 [M+H]^+^. The MS^2^ spectrum of oleamide displayed mass peaks at *m*/*z* 265 and 247, corresponding to neutral losses of ammonia and water, respectively. In MS^3^, a mass peak at *m*/*z* 247 was observed upon fragmentation of the molecular ion at *m*/*z* 265 (water loss), while further fragmentation of *m*/*z* 247 showed losses of 14 *m*/*z*, corresponding to -CH2- cleavages, indicative of acyl chain lipids [33]. These data are in line with our previously reported results [24].

**Figure 1:**
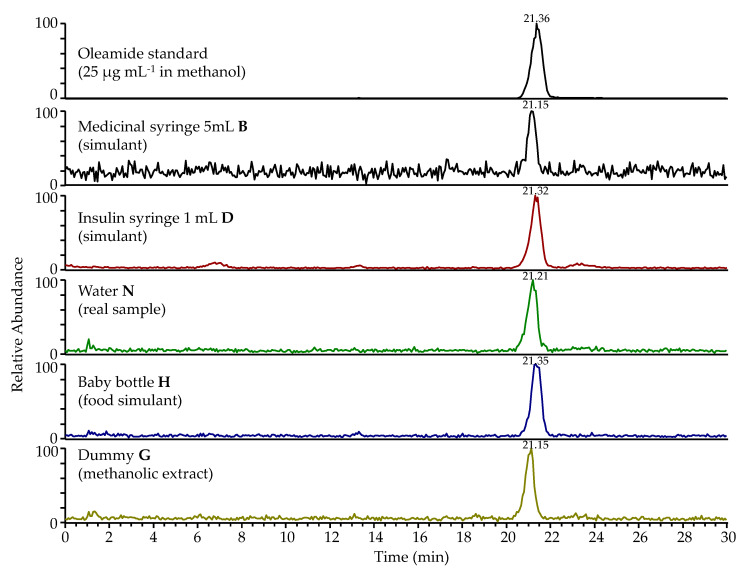
Selected ion monitoring (SIM) chromatograms of oleamide standard and some of the tested extracts, as shown in Table 1.

SIM mode was used for quantification purposes (Figure 1 from the current manuscript; Reference [24] reports only qualitative data) using two quadratic calibration curves. The first calibration curve included five standard concentrations in a lower μg mL^−1^ concentration range (0.098, 0.195, 0.391, 0.781, 1.563). The second calibration curve included five standard concentrations in a higher μg mL^−1^ concentration range (1.563, 3.125, 6.25, 12.5, 25). R^2^ for the first and the second curve were 0.9996 and 0.9983 respectively, LOD and LOQ for oleamide were 0.049 μg mL^−1^ and 0.098 μg mL^−1^ respectively, and RSD% of oleamide area at 25 μg mL^−1^ (*n* = 6) was 1.0%. Accuracy of the method measured at levels 0.391 µg mL^−1^ and 6.25 µg mL^−1^ was 97% and 98% respectively, while precision calculated for the areas of vitamin water samples and oil samples (non-exposed to contact conditions) in six replicates was 4.8% and 5.4%, respectively.

Surprisingly, HPLC glass vial inserts used at first during the LC-MS analysis hindered the quantification of oleamide, due to oleamide-leaching [24]. Therefore, the quantification study was repeated with other pre-tested and oleamide-free HPLC glass vial inserts.

### 3.2. Medicinal Plastic Materials

Oleamide is considered a biologically active compound, inducing a variety of pharmacological effects, as briefly described in the introduction. Therefore, besides labware extracts [24], plastic contact materials used in medicine and food, which were supposed to contribute to oleamide human daily intake, were extracted and tested (Table 1). 

The presence of oleamide in the extract was not related to the producer of the tested consumer products (brands A–E, Table 1) or type of a polymer. Namely, methanolic extract of one brand (brand B) of 5 mL syringes (also available as laboratory syringe for filtration) contained a high amount of oleamide (7351 ng mL^−1^), which was not the case with methanolic extract of insulin syringes of the same brand (<LOQ). On the contrary, methanolic extract of insulin syringes obtained from another brand (brand D) had a comparable chromatographic profile to that of syringe (brand B) methanolic extract and showed an even higher concentration of oleamide (21,984 ng mL^−1^). The water (formulation simulant) extract of this type of insulin syringe possessed 152 ng mL^−1^ of oleamide (Table 1). This concentration is estimated to leach into an aqueous insulin formulation which would consequently be administered parenterally. Taking these results into account, a person with insulin therapy, using 1 mL insulin syringe of brand D, can co-administer 0.15 µg oleamide/injection. Parenteral administration of a drug, using a medicinal syringe of brand B, contributes to oleamide intake of 0.06 μg/syringe. The estimated value of oleamide’s parenteral intake, when administering an infusion using a 250 mL infusion bottle, is 4 μg per bottle.

Since the amount of oleamide in the 26th rinsing extract of a 5 mL plastic syringe using methanol was found to be 513 ng mL^−1^, pre-washing of the syringes before use (including filtration in analytical laboratories) would not help to wash out the total amount of oleamide present in the polymer.

Oleamide introduced into the human body is further metabolized *in vivo* to oleic acid by an integral membrane enzyme, called fatty acid amide hydrolase [17], however to an unknown extent.

### 3.3. Baby Bottles and Plastic Food/Beverages Containers

Baby bottles (Table 1) filled with 50% ethanol_(aq)_ as a food (milk) simulant were exposed to a shorter contact time [32] in comparison to other milk bottles. This is reasonable, as infant formula spends less time in contact with the plastic container than the commercial bottled milk. Since baby bottle dummies (made of silicon) were extracted by methanol for the preliminary studies, further extraction with a simulant was avoided, assuming that the largest amount of oleamide had already been extracted by methanol. The concentration of oleamide leached in infant formula (milk) simulant from baby bottles (made of polypropylene) ranged from 12 to 23 ng mL^−1^, while methanol extracts of dummies contained from 30 to 39 ng mL^−1^ (Table 1). The estimated human intake of oleamide per volume of a baby bottle was also calculated and ranged from 3 µg to 6 µg per bottle (Table 1). Since baby bottles were of different volumes, these values were re-calculated estimating intake of 1−2 μg of oleamide per each decilitre of infant formula consumed. Oleamide leaching from polypropylene baby bottles was already reported previously [10].

Among the plastic food and beverage containers, carbonated soft drinks, vitamin water, milk, yogurt, oil, and water bottles, filled with food simulants or real samples, were tested without exposure to contact conditions or after exposure to contact conditions (Table 1). Water bottles obtained from different brands were exposed to contact conditions, prescribed for long-term exposure of liquid content to plastic material (Section 2.3). Although 10% ethanol_(aq)_ solution could be used as a simulant [32], the original bottled water was not replaced due to the simple matrix and was tested as a real sample. For bottles of carbonated soft drinks, vitamin water, milk and liquid yogurt, food simulants [32] were preferred due to the complexity of the original liquid matrices. 

Two identical vegetable oil bottles were tested with the original content, considered as both a food simulant [32] and a real sample at the same time (Table 1). Only one of the oil bottles (label ‘FS’, Table 1) was exposed to contact conditions. An additional milk bottle containing the original content was also tested. For milk and oil bottles containing the original content, LLE for purification of oleamide extract from complex matrices was performed (Section 2.3 and Table 1). To obtain the final oleamide concentrations, the calculated recoveries of oleamide from oil not exposed to contact conditions (50.7%), oil exposed to contact conditions (53.9%) and milk (22.3%) matrices were taken into consideration. A possible explanation for the low recovery values, especially in the case of milk, might be the potential oleamide loss caused by freezer-accelerated lipid precipitation following LLE (Section 2.3).

As expected, the concentration of oleamide in the extract of oil exposed to contact conditions (238 ng mL^−1^) was slightly higher than in the extract of oil not exposed to contact conditions (217 ng mL^−1^), although the concentration in both cases was relatively high. These numbers may be comparable due to the possibility that the major amount of oleamide was already leached in the oil before executing the aging procedure. Due to its lipophilic character, the oil may stimulate the diffusion of slip additives from plastics to a large extent [2]. However, oleamide, as a derivative of oleic acid, is also known to be naturally present in plant materials [34], which may also include vegetable oil. Interestingly, a much higher concentration of oleamide was obtained for the real milk sample (293 ng mL^−1^) than for its simulant (3−9 ng mL^−1^). This discrepancy could be explained with the natural presence of oleamide in milk [34], as a biological fluid or with the extraction potential of milk itself. Namely, milk might have extracted the majority of oleamide from the polymer already, before the bottle was emptied and subsequently exposed to the simulant. To discard the later doubt, an empty, clean and non-pre-filled bottle, dedicated for milk storage, should be obtained from the producer for testing purposes. Liquid yogurt simulants contained oleamide in a concentration of 17 ng mL^−1^, vitamin water simulant contained 15 ng mL^−1^, carbonated soft drink simulants showed concentrations in the range of 6−15 ng mL^−1^ and oleamide in bottled water was present in the range of 11−18 ng mL^−1^ (Table 1). The human intake of oleamide per volume (mL) of tested food and beverages stored in a plastic container was also estimated and is shown in Table 1. These numbers were re-calculated for 500 mL of bottled liquid and the estimated oleamide intake when calculated through simulant ranged from 2 to 9 μg (milk: 2−5 μg, liquid yogurt: 8−9 μg, water: 6−9 μg, soft drinks including vitamin water: 3−8 μg), while for real matrices (oil/milk) it was in the range from 109 to 147 μg. The latter numbers can be explained by the possible presence of oleamide in natural matrices [34] or by the extraction power of these lipid matrices, as already explained above. The measured values are, according to the current EU regulations, considered of no concern as oleamide is listed in the European Commission’s document without a specific migration limit (SML) [32].

Although all tested food/beverages containers, except baby bottles, were made of polyethylene terephthalate (PET) polymer, which should be free of oleamide [35], leaching of oleamide was detected from all of them (Table 1). According to some published literature, the presence of oleamide may be a consequence of the use of a lubricant coating for the cap sealing, applied for easier opening [36,37], or may be introduced into the liquid through some other sources, such as water/liquid processing steps (transport pipelines, pipes, storage tanks, bottling process, filtering systems, disinfection) [36]. Recently, oleamide was found to leach from reusable PET bottles into different water and liquid food samples as well [38]. Erucamide, another fatty acid amide lubricant, which is not a standard additive of PET polymer [35], was also found in mineral water stored in PET containers in concentrations ranging from 2 to 182 ng mL^−1^ [39]. Therefore, additional experiments to identify the source of oleamide (e.g., containers, closures, other parts) were performed (Section 3.4). Brands of the tested marketed products (brands F–T) were shown to be unrelated to oleamide leaching. This is reasonable for the manufacturers of food and beverages (brands I–T), as they are most probably buying plastic containers from other companies, specialized for producing plastic packaging. Moreover, there is a high possibility that they change the suppliers of plastic containers from time to time. Therefore, batch-to-batch reproducibility should be questioned as well.

Although hydrolysis of oleamide in simulated gastric fluids was found to be negligible, the addition of ‘bile salts’ to the simulant significantly increased its degree of hydrolysis to about 95% and promoted the formation of oleic acid, described as a harmless substance [40]. On the other hand, few case studies, showing the irritant activity of oleamide [41,42], exist. Another recent study reports the cytotoxic activity of oleamide based on ToxCast software data [29]. Moreover, oleamide was classified into class III (highly toxic compound), according to Cramer rules and Toxtree software [25,27,30]. According to the decision tree proposed by The International Life Sciences Institute (ILSI), if the limit of 90 µg/person/day for a class III compound is exceeded, ‘risk assessment requires compound-specific toxicity data’ [43,44]. However, ToxCast and Toxtree information obtained for oleamide are based only on in silico data. Due to the limited studies investigating the toxicological effects of oleamide, read-across from erucamide (similar structural, physico-chemical and toxicokinetics properties), which is concluded to exhibit low toxicity, can be applied to address this data gap [45]. However, future extensive toxicological studies on long-term exposure to oleamide might decrease the uncertainty in the evaluation of human health risk.

### 3.4. Identification of the Source of Migrated Oleamide

According to some published literature (as already described in Section 3.3), the presence of oleamide in PET bottles may be attributed to a lubricant coating applied to the closure systems, enabling easier opening [36,37], or to some liquid processing steps [36]. Therefore, to identify the source of oleamide, the separate components of the items that showed migration of oleamide above LOQ (Table 1) were tested for its presence. Oleamide was analyzed as extractable, using methanol as an extraction solvent to test each item component (non-exposed to contact conditions) separately. As presented in Table 2, both syringes parts (barrel and plunger) showed the presence of oleamide. The manufacturer of the syringe of brand B has specified oleamide as a plunger lubricant only. However, oleamide may have migrated from the plunger to the barrel, as these two components are in tight contact throughout the storage time. Baby bottle containers and the corresponding dummies tested separately also resulted as positive for the presence of oleamide (Table 2), as it is also clear from Table 1. Baby bottles were designed in a way that other plastic parts could not come into contact with the liquid food. All other containers and closures belonging to *i.v.* infusion and food/beverages, tested separately, contained oleamide as well (Table 2).

### 3.5. Screening of Other Extractables and Leachables

Besides oleamide, other contaminants were also observed in the chromatograms of the analyzed extracts. Some of the frequently occurring contaminants in the LC-MS chromatograms were tentatively identified based on the literature data (Table 3). In the LC-MS chromatograms of the extracts, obtained from one type of 5 mL plastic medicinal syringes and one type of 1 mL insulin syringes, individual mass peaks at m/z 254 (unsaturated amine), m/z 280 (unknown), m/z 256 (C14 unsaturated mono alcohol derivative) and m/z 228 (unknown) were tentatively identified as related impurities of oleamide [35]. Some plastic materials (e.g., plastic bottles for water) can also leach different epoxides or aldehydes as products of oleamide double-bond oxidation, following bottle sterilization with ozone or bottle exposure to ultraviolet light [37], although their presence was not demonstrated in the analyzed extracts and leachates by the current method. A compound eluting at retention time (t_R_) = 23.77 was tentatively identified as elaidamide (Table 3), a trans isomer of oleamide. Unequivocal identification of these compounds is, however, out of the scope of our paper, and it could be a subject of a future study.

## 4. Conclusions

Leaching of a known polymer lubricant and bioactive compound, oleamide, from food/beverage and medicine plastic contact materials into polymer contact liquid was studied. An UHPLC-MS method was developed and applied to identify and quantify oleamide using HESI in positive ion mode. Almost all of the tested containers (all food/beverage and some medicine containers), regardless of the manufacturer or type of a polymer, leached oleamide, which was further quantified in SIM mode. Moreover, intake of oleamide per volume of tested food/beverage or medicine material was estimated. The sources of oleamide migration (e.g., barrels, plungers, containers, closures, dummies) were also identified. As oleamide is listed in the current EU regulations without a specific migration limit, the reported values are considered of no concern for the customers. However, toxicological studies investigating the health effects of oleamide are limited. In addition, other extractables and leachables were tentatively identified in the tested extracts and leachates respectively, which can be the subject of another detailed study in the future.

## Figures and Tables

**Table 1:** Disposable medicinal and food/beverage containers tested for oleamide leaching by the LC-MS method together with the estimated intake of oleamide per volume of the tested material. Data for the concentration of oleamide in extraction media represent means ± standard deviation (SD) (*n =* 3); paired t-test with * *p* < 0.05; ** *p* < 0.01; *** *p* < 0.001 compared to blank.

Material Tested with LC-MS Method	Type of a Polymer	Extraction Media/Type of Simulant	Contact Conditions	Pre-treatment before Analysis	Presence of Oleamide Peak in SIM (*m/z* 282)	Concentration of Oleamide in Extraction Media (ng mL^−1^)	Estimated Intake of Oleamide (µg) Per Volume (mL) of the Tested Material
**Medicinal plastic material**							
Intravenous infusion bottle **A**	LDPE	S (EtOH10)	10 days, 60 °C	conc.	+	17 ± 1 ***	4/250
Syringe 5mL **B**	PP + PE	MeOH		non conc.	+	7351 ± 1963 *	/
Syringe 5mL **B**	PP + PE	S (H_2_O)		conc.	+	12 ± 3 **	0.06/5
Syringe 5mL **C**	PP + PE	MeOH		non conc.	-	<LOQ	/
Syringe 5mL **C**	PP + PE	MeOH		conc.	+	<LOQ	/
Syringe 5mL **C**	PP + PE	S (H_2_O)		conc.	-	<LOQ	/
Insulin syringe 1 mL **D**	PP	MeOH		non conc.	+	21,984 ± 1155 ***	/
Insulin syringe 1 mL **D**	PP	S (H_2_O)		conc.	+	152 ± 8 ***	0.15/1
Insulin syringe 1 mL **B**	LDPE	MeOH		non conc.	-	<LOQ	/
Insulin syringe 1 mL **B**	LDPE	MeOH		conc.	-	<LOQ	/
Insulin syringe 1 mL **B**	LDPE	S (H_2_O)		conc.	-	<LOQ	/
Enteral syringe **E**	PP	MeOH		non conc.	-	<LOQ	/
Enteral syringe **E**	PP	MeOH		conc.	+	<LOQ	/
Enteral syringe **E**	PP	FS (EtOH50)		conc.	-	<LOQ	/
**Baby bottles**							
Baby bottle **F**	PP	FS (EtOH50)	2 h, 40 °C	conc.	+	23 ± 3 **	3/125
Baby bottle **G**	PP	FS (EtOH50)	2 h, 40 °C	conc.	+	12 ± 2 **	3/250
Baby bottle **H**	PP	FS (EtOH50)	2 h, 40 °C	conc.	+	20 ± 2 **	6/300
Dummy **F**	silicon	MeOH		conc.	+	33 ± 8 **	/
Dummy **G**	silicon	MeOH		conc.	+	30 ± 4 **	/
Dummy **H**	silicon	MeOH		conc.	+	39 ± 9 **	/
**Plastic food containers**							
Carbonated soft drink **I**	PET	FS (AC3)	10 days, 60 °C	conc.	+	15 ± 3 **	8/500
Carbonated soft drink **J**	PET	FS (AC3)	10 days, 60 °C	conc.	+	6 ± 1 **	9/1500
Vitamin water **K**	PET	FS (AC3)	10 days, 60 °C	conc.	+	15 ± 3 **	8/500
Milk (3.5% fat) **L**	PET	FS (EtOH50)	10 days, 20 °C	conc.	+	3 ± 2 *	3/1000
Milk (3.5% fat) **M**	PET	FS (EtOH50)	10 days, 20 °C	conc.	+	9 ± 5 *	9/1000
Milk (1.5% fat) **N**	PET	FS (EtOH50)	10 days, 20 °C	conc.	+	8 ± 3 *	8/1000
Liquid yogurt (4% fat) **O**	PET	FS (EtOH50)	10 days, 20 °C	conc.	+	17 ± 3 **	9/500
Liquid yogurt (1.3% fat) **P**	PET	FS (EtOH50)	10 days, 20 °C	conc.	+	17 ± 2 **	8/500
Oil **N**	PET	FS (oil = RS)	10 days, 60 °C	LLE + conc.	+	238 ± 21 **	238/1000
Milk (3.5% fat) **M**	PET	RS (milk)		LLE + conc.	+	293 ± 47 *	293/1000
Oil **N**	PET	RS (oil)		LLE + conc.	+	217 ± 9 ***	217/1000
Water **Q**	PET	RS (water)	10 days, 60 °C	conc.	+	16 ± 2 **	8/500
Water **R**	PET	RS (water)	10 days, 60 °C	conc.	+	11 ± 1 ***	6/500
Water **S**	PET	RS (water)	10 days, 60 °C	conc.	+	12 ± 3 **	6/500
Water **G**	PET	RS (water)	10 days, 60 °C	conc.	+	12 ± 2 **	6/500
Water **T**	PET	RS (water)	10 days, 60 °C	conc.	+	18 ± 4 **	9/500
Water **N**	PET	RS (water)	10 days, 60 °C	conc.	+	14 ± 2 **	7/500

S: simulant (formulation simulant); FS: food simulant; RS: real sample (the original content of the container); conc.: concentration of the extract; non conc.: extract that was not concentrated; LLE: liquid-liquid extraction; LDPE: low-density polyethylene; PP: polypropylene; PE: polyethylene; PET: polyethylene terephthalate; EtOH10: 10% ethanol(aq); EtOH50: 50% ethanol(aq); AC3: 3% acetic acid(aq); MeOH: methanol; *m/z*: mass-to-charge ratio; -: not detected; +: detected; /: estimation of oleamide intake was not calculated; either it was not possible (concentration of oleamide < LOQ) or the extraction solvent did not simulate the real container content; <LOQ: below the limit of quantification; A–T: labels representing different brands per tested material; d: days; h: hours.

**Table 2:** Material parts tested for oleamide migration.

Material Tested	Tested Parts	
	barrel	plunger
Syringe 5 mL **B**	+	+
Insulin syringe 1 mL **D**	+	+
	container	dummy
Baby bottle and dummy **F**	+	+
Baby bottle and dummy **G**	+	+
Baby bottle and dummy **H**	+	+
	container	closure
Intravenous infusion bottle **A**	+	+
Carbonated soft drink **I**	+	+
Carbonated soft drink **J**	+	+
Vitamin water **K**	+	+
Milk (3.5% fat) **L**	+	+
Milk (3.5% fat) **M**	+	+
Milk (1.5% fat) **N**	+	+
Liquid yogurt (4% fat) **O**	+	+
Liquid yogurt (1.3% fat) **P**	+	+
Oil **N**	+	+
Water **Q**	+	+
Water **R**	+	+
Water **S**	+	+
Water **G**	+	+
Water **T**	+	+
Water **N**	+	+

+ detected; A,B,D,F–T: labels representing different brands per tested material.

**Table 3:** Tentative identification of some frequently occurring contaminants, observed in the LC-MS chromatograms of the analyzed extracts.

*m/z* (ESI^+^)	Tentative Identification	*t_R_* (min)	Material	Reference
228	*N*-butyl-*p*-toluenesulphonamide; unknown (present in oleamide standard); myristamide	8.44	Insulin syringe (1 mL), plastic syringe (5 mL)	[33,35,46]
242	Tetrabutylammonium cation	11.03, 12.16	Plastic syringe (5 mL)	[47]
245	Oxotris(propan-2-olato)vanadium; 2,2’-dihydroxy-4-methoxybenzophenone; 1,1’-azobis(cyclohexanecarbonitrile); 2,2’-dimethoxy-4,4’-benzidine;	8.45	Insulin syringe (1 mL), plastic syringe (5 mL)	[46]
250	Triallyl (iso)cyanurate	9.04	Dummy	[46]
254	Unsaturated amine (present in oleamide standard)	10.37	Insulin syringe (1 mL), plastic syringe (5 mL)	[35]
256	C_14_ Unsaturated mono alcohol derivative (present in oleamide standard); palmitamide	18.41	Insulin syringe (1 mL), plastic syringe (5 mL)	[33,35]
268	?	14.89	Insulin syringe (1 mL), plastic syringe (5 mL)	[48]
273	Tetrapropylene glycol [M+Na]^+^; monomethoxytrityl cation	18.41	Insulin syringe (1 mL), plastic syringe (5 mL)	[47,49,50]
278	2-Ethylhexyl-4-(dimethyl amino) benzoate; ethyl 2-cyano-3,3-diphenylacrylate	8.97	Plastic syringe (5 mL)	[46]
280	Unknown (present in oleamide standard) - oxidation product?	13.49, 14.89	Insulin syringe (1 mL), plastic syringe (5 mL)	[35]
282	*Trans*-oleamide (elaidamide)	23.77	Insulin syringe (1 mL), plastic syringe (5 mL)	[51]
284	Oleamide contaminant; stearamide;	6.05, 21.07	Plastic syringe (5 mL)	[33,35,47,48]
284	Oleamide contaminant; stearamide;	6.05	Food container	[33,35,47,48]
301	Oleoyl chloride; dibutylphthalate [M+Na]^+^; sodium (C_10_-C_18_) alkyl sulfonate; (nonylphenyl)phosphate	6.05, 21.07	Plastic syringe (5 mL)	[35,47,49,51]
310	?	11.03	Plastic syringe (5 mL)	
314	Ethyl stearate; styrene trimer	5.54	Food container, baby bottle, dummy	[35]
331	Pentadecyl sodium sulfate; pentapropylene glycol [M+Na]^+^; dicyclohexyl phthalate	23.19	Baby bottle, food container	[35,47,49]
341	Glycerol monostearate [M+H-H_2_O]^+^; unknown	18.54	Baby bottle	[35]
348	?	23.19	Baby bottle	
359	Heptadecyl sodium sulfate	18.54	Baby bottle	[35]
362	2-Ethylhexyl 2-cyano-3,3-diphenylacrylate; 2-(4-dodecylphenyl)indole; didecyldimethylammonium chloride	9.04	Dummy	[46]
368	*N,N,N*-Trimethyldocosan-1-aminium cation	8.97, 10.43	Plastic syringe (5 mL)	[47,49]
369	2,2’-(1,4-phenylene) bis[4H-3,1-benzoxazin-4-one]; *N*-[2-[(2-hydroxyethyl)amino]ethyl]oleamide; 2,2’-methylene bis(4-ethyl-6-tert-butylphenol); lignoceric acid; 2-ethylhexyl palmitate	23.19	Baby bottle	[46]
371	Decamethylcyclopentasiloxane; pentasiloxane; octaethylene glycol; dioctyl adipate; bis(2-ethylhexyl) adipate	6.40	Food container, baby bottle, dummy	[47,48,49,51]
376	1,3,5-Tris (2,2-dimethylpropionylamino)-benzene; sodium 3-[(4-anilinophenyl)diazenyl]benzenesulfonate (Acid Yellow 36)	18.54	Baby bottle	[46]
379	α-Cyano-4-hydroxycinnamic acid [M_2_+H]^+^	9.04	Dummy	[47,49]
385	Bisphenol F bis(2-chloro-1-propanol) ether	6.31	Food container, baby bottle, dummy	[46]
397	?	18.54	Baby bottle	[47,49]
399	Setoglaucine (Basic Blue 1); tris-(2-butoxyethyl) phosphate; decyl octyl adipate	4.25, 13.29	Dummy	[46]
403	Acetyltributyl citrate; glyceryl monooleate [M+H+CH_2_O_2_]^+^; unknown	5.54	Food container, baby bottle, dummy	[34]
426	2-(4,6-Diphenyl-1,3,5-triazin-2-yl)-5-(hexyloxy)phenol	20.93, 23.59	Infusion bottle, food container, baby bottle, dummy	[46]
431	2,5-Bis(5’-tert-butylbenzoxazol-2-yl)thiophene	12.03	Dummy	[35,48]
455	?	8.45	Plastic syringe (5 mL)	[47,49]
456	?	20.93–23.00	Infusion bottle, food container, baby bottle, dummy	
463	Septapropylene glycol [M+K]^+^;	18.50	Dummy	[49]
480	?	18.50	Dummy	
485	?	2.19	Food container	[47,49]
485	?	2.19, 10.76	Baby bottle	[47,49]
486	?	2.26, 4.65	Infusion bottle	
491	Bisphenol A diglycidyl ether; derivative	18.50, 21.60	Dummy	[46]
502	?	9.19	Baby bottle	
511	1-Piperidinyloxy, 4,4’-[1,10-dioxo-1,10-decanediyl)bis(oxy)]bis[2,2,6,6-tetramethyl]	17.80	Plastic syringe (5 mL)	[46]
563	4,4’-Bis(2-sulphostyryl)biphenyl, disodium salt	21.00	Insulin syringe (1 mL), plastic syringe (5 mL)	[46]
567	Irganox 1330 fragment (2,6-di-tert-butylphenol split off)	4.25	Dummy	[52]
740	?	4.39	Infusion bottle	
953	?	7.91	Infusion bottle	[47]
955	?	4.45, 7.91	Infusion bottle	

*m/z*: mass-to-charge ratio; *t_R_*: retention time; ?: unknown identity.
